# Challenges in software applications for the cognitive evaluation and stimulation of the elderly

**DOI:** 10.1186/1743-0003-11-88

**Published:** 2014-05-15

**Authors:** Sandra Rute-Pérez, Sandra Santiago-Ramajo, María Visitación Hurtado, María José Rodríguez-Fórtiz, Alfonso Caracuel

**Affiliations:** 1 Clinical Neuropsychology Research Group, University of Granada, Granada, Spain; 2 International Research, University of La Rioja, La Rioja, Spain; 3 Software Engineering Department, University of Granada, Granada, Spain; 4 CITIC: Research Center for Information and Communication Technologies, University of Granada, Granada, Spain; 5 Developmental and Educational Psychology Department, University of Granada, Granada, Spain; 6 CIMCYC: Mind, Brain and Behavior Research Center, University of Granada, Granada, Spain

**Keywords:** Application software, Software design, Cognitive evaluation, Cognitive stimulation, Aging

## Abstract

**Background:**

Computer-based cognitive stimulation applications can help the elderly maintain and improve their cognitive skills. In this research paper, our objectives are to verify the usability of PESCO (an open-software application for cognitive evaluation and stimulation) and to determine the concurrent validity of cognitive assessment tests and the effectiveness of PESCO’s cognitive stimulation exercises.

**Methods:**

Two studies were conducted in various community computer centers in the province of Granada. The first study tested tool usability by observing 43 elderly people and considering their responses to a questionnaire. In the second study, 36 elderly people completed pen-and-paper and PESCO tests followed by nine cognitive stimulation sessions. Meanwhile, a control group with 34 participants used computers for nine non-structured sessions.

**Results:**

Analysis of the first study revealed that although PESCO had been developed by taking usability guidelines into account, there was room for improvement. Results from the second study indicated moderate concurrent validity between PESCO and standardized tests (Pearson’s *r* from .501 to .702) and highlighted the effectiveness of training exercises for improving attention (*F* = -4.111, *p* < .001) and planning (*F* = 5.791, *p* < .001) functions.

**Conclusions:**

PESCO can be used by the elderly. The PESCO cognitive test module demonstrated its concurrent validity with traditional cognitive evaluation tests. The stimulation module is effective for improving attention and planning skills.

## Background

Active aging is the process of optimizing opportunities for health, participation and security in order to enhance quality of life as people get older. The word “active” refers to continuing social, economic, cultural, spiritual and civic participation and not just the ability to be physically active or participate in the labor force [1]. A great deal of research has highlighted the important interdependence between the cognitive skills (attention, memory, language, reasoning, visuo-spatial, executive, etc.) and active aging of the elderly [2]. The largest challenge facing the elderly is to combat dementia and maintain cognitive skills [3]. Cognitive decline affects how people perform their *Activities of Daily Living* (ADL), which are essential for independent living. The ADLs which are most quickly affected by cognitive impairment are *instrumental* activities (IADL). These activities include the ability to make decisions and to carry out tasks requiring more complex interactions with their surroundings (cooking and household chores, shopping, using public transport, handling money, managing medication, using the phone, etc.) [4]. Cognitive stimulation programs have proved effective for delaying ADL deterioration and consequently the onset of dementia [4,5]. There are, however, important drawbacks associated with standard face-to-face stimulation programs such as the cost of serving an extremely large and ever increasing population. This enormous challenge may be tackled with computer-based cognitive stimulation programs. Willis et al. [6] found that computer-based reasoning training results in long-term IADL maintenance. Despite the large scope of this finding, there remains the question of how should the challenge of universally extending the use of computers among the elderly be met. There are various key factors and these include usability, motivation, validity and universally free distribution.

It had already been suggested back in the 1970s that human factors research should design for older people by considering the deterioration that accompanies aging [7]. As people age, there is a reduction not only in their general ability to maintain attention but also in their sensory motor skills. It is therefore desirable to adapt cognitive software applications in order to improve their *usability* and *accessibility*. The International Organization for Standardization (ISO) defines *usability* as “the extent to which a product can be used by specified users to achieve specified goals with effectiveness, efficiency and satisfaction in a specified context of use”. This includes methods for improving ease-of-use during the design process. *Accessibility*, on the other hand, is “the degree to which a product, device, service or environment is available to as many people as possible” [8,9]. Assistive and adaptive technology enables a person to complete an otherwise impossible task, thereby enabling the elderly and the disabled to live more independently and participate more fully in society.

The main limitations of the elderly in terms of health that must be considered when using a computer are [10,11]:

• Sight: reduction in the field of vision, ability to distinguish small details, process visual information and adjust to darkness

• Hearing: reduction in the ability to hear certain sound timbres or distinguish certain frequencies

• Mobility: slower response times, reduction in fine motor skills and greater fatigue

• Cognitive: decrease in attention span, especially if there are distractions, short-term and working memory loss.

Unlike younger generations, older people have less experience or are afraid of using computers and this results in the following difficulties [12,13]: they take longer to perform certain activities and to read instructions and textual information, they make more mistakes, they forget the point of the activity they are performing, they are more often confused by or do not understand technical language, they are reluctant or refuse to do something they think will cause system failure, and they get more upset and often blame themselves if something goes wrong.

As well as being usable and accessible, programs should provide specific motivation for a population that both fears and rejects the computer. Factors, therefore, such as constant guidance through virtual peer models, feedback after valid cognitive skills assessment, encouragement after each exercise and gradual grading of the difficulty of activities may help keep the elderly motivated [14].

In 2010, Owen et al. wrote in Nature that “the widely held belief that commercially available computerized brain-training programs improve general cognitive function in the wider population in our opinion lacks empirical support” [15]. Validation of assessment tools and evidence of the effectiveness of training exercises was therefore necessary before sitting people in front of a computer.

One last idea for addressing the challenge is to attempt to develop a universal, free, open-source software application that may easily be adapted to different user profiles, languages and cognitive evaluation and stimulation requirements.

This paper had three main objectives: the first was to check recommendations and design solution guidelines and explore feedback in order to improve the usability of an open-software application, which we have called PESCO (from *Programa de EStimulación COgnitiva* in Spanish), for cognitive stimulation and evaluation; the second was to check the concurrent validity of tests for cognitive assessment of PESCO; and the third was to determine the effectiveness of training activities designed for cognitive stimulation.

## Method

### Software application

PESCO has been used both for evaluation and stimulation. It is an open-source Linux software application for cognitive stimulation in the elderly funded by a public regional body and which the authors of this study developed in 2011. The application is currently used in Guadalinfo Centers, which are local authority community computer centers in Southern Spain (http://www.guadalinfo.es/centros), and is available for download from the PESCO website (http://asistic.ugr.es/pesco/).

PESCO includes a series of tests for assessing cognitive status and exercises for training the cognitive skills which are believed to be linked to the early detection or delay of dementia: attention span, memory, reasoning and planning [6,16-18]. The exercises have different levels of difficulty, support and encouragement in order to improve adaptation to cognitive baseline status and motivation.

PESCO automatically records the time taken to perform each activity and the success and failure rate. Once participants have successfully completed 80% of the PESCO items, they proceed to the next level and are given some form of reward.

PESCO has one two-session module for baseline assessment of subjects in four cognitive areas: two attention tests (Numbers, Pyramids), two memory tests (List of Words, Number-Vowel Sequencing), two reasoning tests (Series of Semantic, Series of Logic) and a planning test (Parcel delivery). Additionally, two questionnaires about ADL performance are included. All cognitive tests could subsequently be used for a post-stimulation assessment session. Parallel versions of the List of Words, Series of Semantic, Series of Logic and Parcel Delivery tests are used in this session. The two versions of the word list were performed with words of similar frequency of use [19]. The versions of the Series of Semantic and Logic were performed by randomizing items of similar difficulty. The two versions of the Parcel Delivery test differed in where the items were located on the screen. Subsequently, one cognitive stimulation module provides systematic learning through training exercises for each cognitive function. The stimulation module has nine sessions, each of which takes between 45 and 60 minutes. It is recommended that these are performed twice a week.

**Figure 1:**
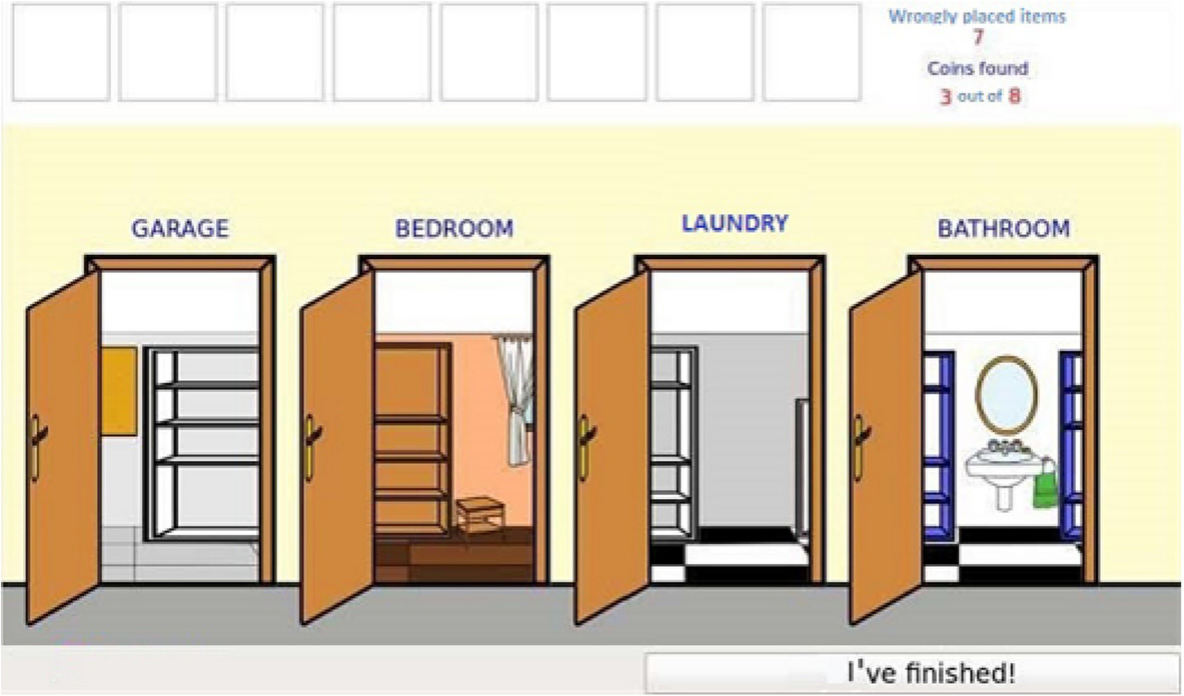
Searching for Objects: screen of the corridor.

**Figure 2:**
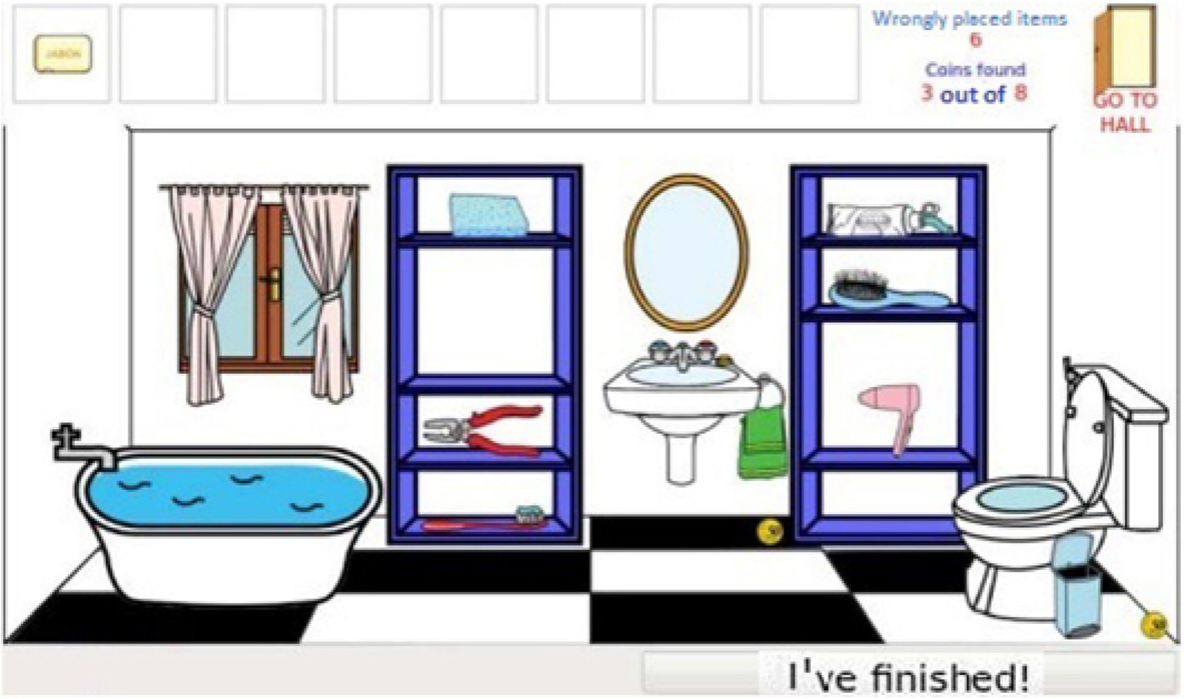
Searching for Objects: screen of a room.

Cognitive stimulation of attention is based on two activities designed to train each relevant attention component. The first is *Balloons*, an n-back task [20] (1-back, 2-back and 3-back) designed with balloons which move from the right to left-hand side of the screen, and then appear and disappear one at a time in order to train both focused and sustained attention and working memory. The second is *Searching for Objects*, an exercise that has been specially designed to improve the user’s sustained, selective and alternating attention. The user must scroll through the different rooms in the house shown in Figure 1. The aim of the game is to find any household object which is in the wrong place and move it to its correct place. Users are also asked to collect the coins they find in each room. An example of one such room is shown in Figure 2. The user must find the objects which are not usually found in that room.

Three different exercises address various aspects of memory stimulation such as working memory, short- and long-term memory by coding, storage and recovery processes. The first is *Lists of Errands*, which is designed to improve verbal learning and episodic memory through strategy instruction and practice. In order to use ADL, the lists comprise common errands that elderly people normally carry out. The second is called the *Bag of Items*, a working memory training exercise based on a simulated walk through a neighborhood, in which the participant exchanges relevant objects in various local places. The last is the *Classifiable Objects* task which is based on semantic and category strategy use for learning new materials.

Reasoning skills are trained through five tasks (*Semantic Analogies, Logic Reasoning, Semantic Reasoning, Visual Reasoning* and *Sorting*) which focus on improving the ability to solve problems containing a serial, semantic or merely visual pattern.

The multitask-based exercise called *Gift Purchase* has been designed to improve planning skills (establishing goals, control implementation and measuring results). The screen shows a shopping area and the participant must buy a series of gifts for other people on account of each person’s listed preferences and within a limited budget.

Three training exercises (*Searching for Objects, Lists of Errands* and *Gift Purchase)* simulate the ecological tasks or surroundings such as the home, neighborhood or shopping centers where users must perform specific everyday activities. Each training exercise has four levels of difficulty and users automatically proceed to the next level as their performance improves. A full explanation and figures for each test and exercise are available on the PESCO website.

This software application was designed following the guidelines proposed by several authors [6-8] and as summarized in Table 1.

**Table 1:** Guidelines for accessibility and usability solutions for applications used by the elderly

**Sight**	High text-background contrast
	Large screens
	No flashing images or text
	High screen resolution
	Avoid the use of quick screens
	Clear, simple screens
	Minimum font size of 10-12pt
	Audio instructions rather than visual instructions
	Easy-to-read font
**Hearing**	Written content as an alternative to audio content
	Possibility of changing frequency and tone
	Possibility of changing the volume
**Mobility**	Separation between selectable objects
	Possibility of using different I/O peripherals
	Use of touch screen
	Moving objects should not be used as cascading drop-down menus
**Cognitive**	Show context information for guidance
	Limit functionality
	Facilitate the use of forms
	Design should be error-free
	Use short texts and images
	Tactile interface
	Assistant to provide guidance
	Use of demos and trial runs
	Use only one font face on the screen
	Limit the amount of information displayed
	Use clear, imperative instructions
	Show short, clear error message
	Encourage work and achievements
	Use of audio encouragement
	Clear, imperative instructions
	Highlight selections

User interfaces were designed to be useable and accessible by the elderly following the proposed guidelines. Two elements were also added in order to motivate the elderly:

• A virtual assistant called Pepe was designed as a model users could identify with to offer guidance and explain the objectives and steps of each exercise. This also provides support and encouragement.

• Medals (gold, silver and bronze) are awarded to users at the end of each exercise to grade their performance. This kind of encouragement stimulates competitiveness and provides a sense of achievement.

**Figure 3:**
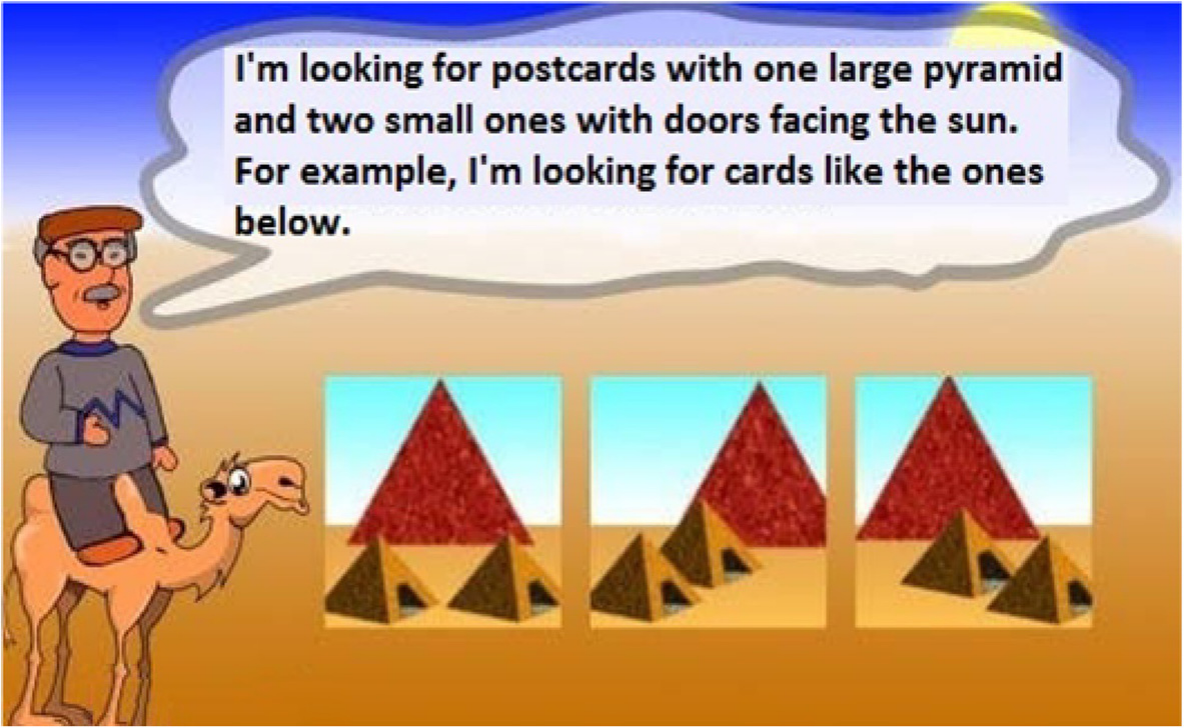
Instructions of the Pyramid Test.

**Figure 4:**
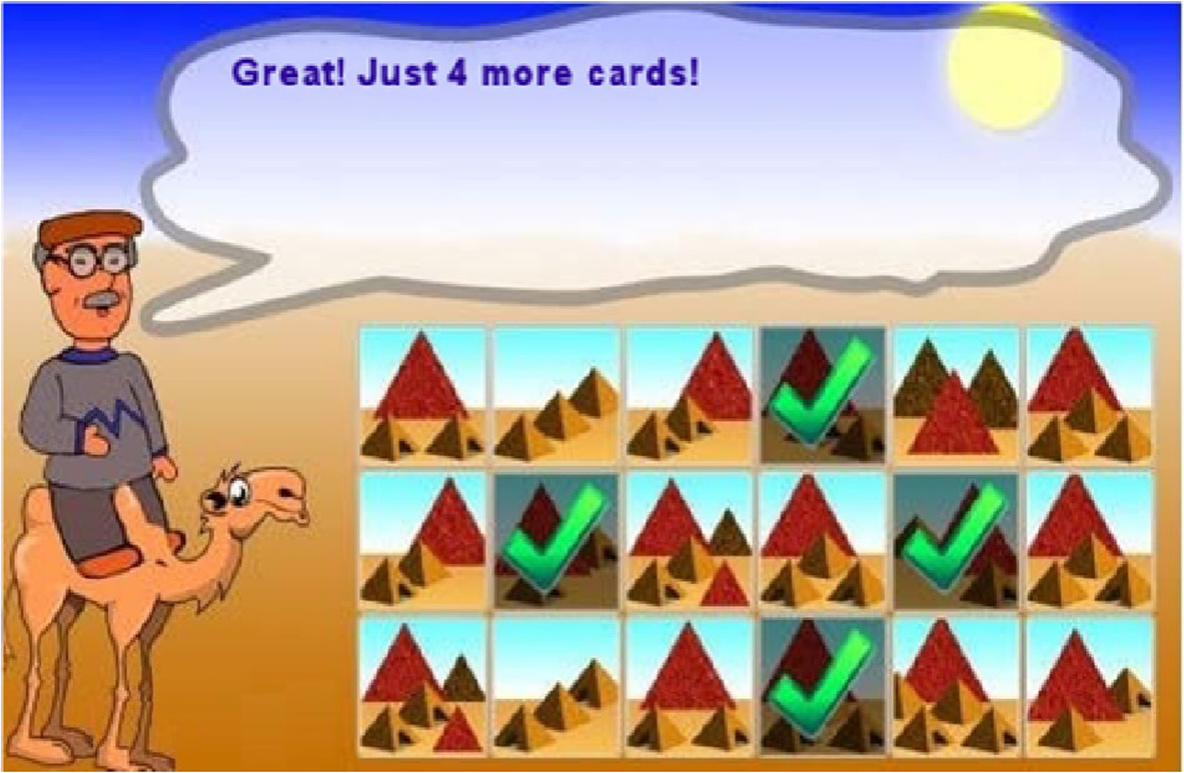
Demo of the Pyramid Test.

**Figure 5:**
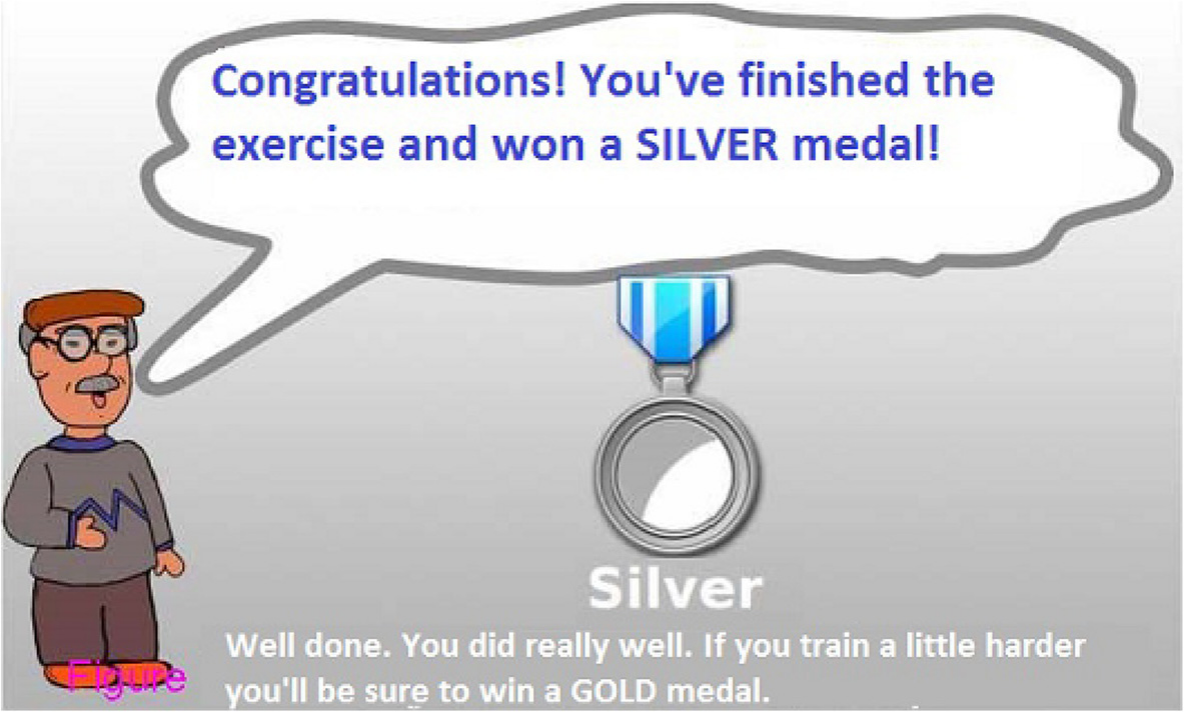
**Feedback screen.** A medal is used to give feedback to the user.

At the beginning of each task, a screen provides instructions for the user (see Figure 3 for an example). A demo then follows in order to check that the user has really understood the test (see Figure 4). Finally, a screen provides the user with feedback about their performance using the incentive of gold, silver or bronze medals as a reward (see Figure 5).

## Method

### Study 1

This study attempted to confirm the hypothesis that PESCO may be used by older generations.

### Participants

Forty-three elderly people (65% women) were recruited from four Guadalinfo centers (three of these in rural areas). The mean age was 74 years (SD = 10.9) and only 35% of the participants had previously used a computer (mean usage of 4.24 hours per week, SD = 2.25).

### Procedure & assessments

In order to assess how PESCO adjusts to usability guidelines, two methods were used: (i) naturalistic observation while participants used the application, and recording of questions, doubts, opinions and spontaneous suggestions about the tool interface and interaction; and (ii) completion of a specifically designed questionnaire to evaluate user satisfaction, expectations and difficulties at the end of each of the twelve PESCO sessions, including cognitive assessment tests and stimulation exercises. All the community centers provided PCs and laptops and accessible computers with special switches and touch screens. Most of the participants interacted with the application using the mouse and only three used the touch screen. Training was supervised by a psychologist and a technician who observed the participants, answered questions, took notes and recorded user suggestions.

### Statistical analysis

The McNemar test was applied to compare the differences between both assessments (baseline and post). Analyses were performed using the statistical package SPSS v19 [21].

### Study 2

In order to achieve our second aim, we conducted a study to (i) validate cognitive tests and (ii) check the effectiveness of cognitive stimulation activities included in PESCO.

### Participants

Two groups of participants were recruited from three Guadalinfo centers (two of these in rural areas). Thirty-six elderly people (55% women), with a mean age of 66 years (SD = 3.7) were recruited for an experimental group involved in test validation and cognitive stimulation through PESCO. New participants were subsequently recruited for comparison (a control group) in order to check the effectiveness of PESCO stimulation. Thirty-four elderly people (67% women), with a mean age of 73 years (SD = 5.8) comprised the control group. Study inclusion criteria were that they should be aged between 60 and 80 years old, possess basic reading skills, score ≥ 21 in the Spanish version of the Mini-Mental State Examination [22], and not have been diagnosed with any cognitive impairment such as mild cognitive impairment, dementia or Alzheimer’s. The MMSE mean was 26.77 for the experimental group (SD = 2.69) and 26.67 for the control group (SD = 2.76). In terms of previous computer experience, 44% of the experimental group and 40% of the control group had used computers before. Samples of Studies 1 and 2 were independent. Both studies were approved by the Ethics Committee on Human Research of the University of Granada. Informed consent and ethical aspects were maintained.

### Procedure & assessments

Subjects in the experimental group were assessed individually in the community computer centers with standard pen-and-paper tests of attention, memory, reasoning and planning (two 1-hour sessions in one week). Data from this assessment were used to determine concurrent validity of the PESCO tests and as a baseline for stimulation. Participants then immediately performed the five cognitive tests included in the PESCO baseline module test to evaluate the same skills (both test types are listed in Table 2). Data protection was guaranteed by the system of the community centers where the study was conducted. Each participant had a username and password to access PESCO. Data was stored on the local computer at the community center and could only be accessed by the person officially in charge of the center.

**Table 2:** Results of experimental group participants in standardized and PESCO tests and correlations between both test types

			***Mean (SD)***	***Pearson’s r***
**Attention**	Standard test	Digit (WAIS-III)	6.73 (2.01)	.573 (*p* < .001)
	PESCO test	**Numbers**	5.09 (1.95)	
	Standard test	d2 test of attention	85.71 (40.09)	.510 (*p* = .005)
	PESCO test	**Pyramids**	61.72 (22.6)	
**Memory**	Standard test	Memory delay of the HVLT	4.03 (2.78)	.656 (*p* < .001)
	PESCO test	**Memory delay of the list of words**	4.73 (3.34)	
	Standard test	*Letter*-*Number* sequencing (WAIS-III)	5.19 (2.33)	.702 (*p* < .001)
	PESCO test	**Number-Vowel Sequencing**	6.36 (2.04)	
**Reasoning**	Standard test	*Similarities* (WAIS-III)	14.53 (4.23)	.552 (*p* = .001)
	PESCO test	**Series of semantic**	4.00 (1.02)	
	Standard test	*Matrix* reasoning (WAIS-III)	9.80 (4.43)	.578 (*p* = .001)
	PESCO test	**Series of logic**	2.41 (1.40)	
**Planning**	Standard test	Zoo map (part 2)	5.97 (3.17)	.501 (*p* = .004)
	PESCO test	**Parcel delivery**	13.68 (1.78)	

HVLT: Hopkins Verbal Learning Test. WAIS-III: Wechsler Adult Intelligence Scale-III. Zoo map: Behavioral Assessment of the Dysexecutive Syndrome Battery. Raw correct test answers were used as variables, except in the case of Parcel Delivery where the number of actions needed was computed (the lower the number, the better the performance). PESCO tests are shown in bold.

The standard pen-and-paper tests applied were the Digit, Letter-Number Sequencing, Similarities and Matrix Reasoning subscales of the Wechsler Adult Intelligence Scale-III (WAIS-III) [23]; the d2 Test of Attention [24]; the Hopkins Verbal Learning Test (Memory delay) [25]; the Zoo map (part 2) subtests of the Behavioral Assessment of the Dysexecutive Syndrome Battery [26]. The group then attended nine cognitive stimulation sessions as scheduled in PESCO (two weekly sessions, each lasting 45-60 minutes). Finally, participants attended the post-stimulation assessment session.

Once the experimental group had completed this procedure, the control group performed the PESCO baseline tests (two sessions in one week), used the community center computers for nine sessions over five weeks (two sessions per week) and attended the post-stimulation PESCO assessment session. During the nine sessions, the control group used a computer for 60 minutes for three different types of task based on the free software available in every Guadalinfo Center: 15 minutes with a standard program such as *Mouse Trainer* or *Keyboard and Mouse Games in Guadalinfo* to improve motor skills, 25 minutes with a LINUX gaming entertainment package where participants were free to choose games such as *Frozen Bubble*, and 20 minutes surfing the web.

### Statistical analysis

Kolgomorov-Smirnov tests supported the normality of the distributions of the main dependent measures. Standardized test scores were compared with those of computerized tests using the Pearson correlation coefficient. Baseline differences between the experimental and the control group were tested with independent-sample t-tests (there were no significant differences in any variable) and the effectiveness of cognitive stimulation was tested using mixed analyses of variance (ANOVAs) 2 (Groups: experimental vs. control) * 2 (Time of evaluation: pre vs. post). All data were analyzed using the SPSS v19 statistical package [21].

## Results

### Study 1

By observing the participants and recording their comments, we discovered that some of the participants found it difficult to read not only the text in certain figures but also long instructions, consequently misunderstanding the instructions and clicking any button (some users tended not to use the buttons that were intended, choosing instead to use other screen elements that were more meaningful to them). Several participants asked for the possibility of listening to the instructions. Most of the participants were looking forward to repeating cognitive stimulation in the coming sessions. There were significant improvements in the variables relating to difficulty, font size/type, instructions being understandable, tasks being enjoyable and tasks being pointless (p < .05). The variable “Screen buttons were difficult to use” was close to significant (p=. 092).

Table 3 shows data taken from the usability questionnaires completed by participants after each assessment (at baseline and post-training times) and the training session.

**Table 3:** Questionnaire results: percentage of positive responses to the items at the end of each session

**Items**	**Baseline assessment**	**Training**	**Post assessment**	***p *** **McNemar test (baseline vs. post)**
1.Tasks were difficult to use	39.5%	20.3%	11.6%	<.001
2.Tasks were frustrating	20.9%	10.4%	14%	.219
3.Screen buttons were difficult to use	25.6%	11%	9.3%	.092
4.Font size/type was unsuitable	23.3%	19.8%	2.3%	.039
5.Instructions or error messages were hard to understand	34.9%	33.8%	11.9%	.008
6.Tasks were not enjoyable	21.9%	22.7%	2.3%	.039
7.Tasks were pointless	21.9%	16.9%	2.3%	.039

Baseline data show the means of the two pre-training assessment sessions (Session 1 and Session 2), training data show the means of the nine training sessions (Session 3 to Session 11) and post-assessment data show the means of Session 12 (post-training).

### Study 2

The results of how the experimental group participants performed in the PESCO tests and corresponding computerized standardized tests can be seen in Table 2. Data from both types of tests indicate that there are moderate and significant correlations between them in the four cognitive areas. The lowest correlation was found between the planning tests (*r* = .501) and the highest correlation was found between the working memory tests (*r* = .702).

Table 4 shows the results of the experimental and the control group in the PESCO tests at baseline and post-stimulation times. The interaction effects between the group and time of assessment were significant in the three PESCO tests: Pyramids (visual attention), Number and Vowel sequencing (working memory) and Parcel Delivery (planning). These results showed a significant improvement by the experimental group in these three cognitive functions.

**Table 4:** Results of ANOVAs of performance in PESCO cognitive assessment tests in the experimental and control groups at baseline and post-stimulation times

**Variables and tests**	**Experimental (n = 36)**		**Control (n = 34)**		**Interaction main effect**	
	**Pre-M (SD)**	**Post-M (SD)**	**Pre-M (SD)**	**Post-M (SD)**	***F***	***p***
Attention						
Numbers	5.09 (1.95)	5.45 (1.07)	5.26 (.56)	5.72 (2.69)	.021	.886
Pyramids	61.72 (22.6)	73.22 (20.7)	68.71 (20.80)	71.33 (21.22)	4.290	.043*
Memory						
Memory delay-list of words	4.73 (3.34)	4.59 (2.99)	4.43 (2.37)	5.23 (1.70)	.097	.757
Number-vowel sequencing	6.36 (2.04)	7.05 (2.65)	5.03 (2.55)	4 (2.38)	4.550	.038*
Reasoning						
Series of semantic	4.00 (1.02)	4.27 (1.03)	3.94 (1.08)	4.36 (1.07)	2.928	.093
Series of logic	2.41 (1.40)	2.77 (1.45)	2.63 (1.34)	2.84 (1.54)	1.253	.268
Planning						
Parcel delivery	13.68 (1.78)	10.68 (2.1)	13.57 (1.9)	13.21 (2.1)	43.682	<.001**

^a^Numbers represent means and standard deviations (in brackets). Explanation of variables: Attention (Numbers: total correct items; Pyramids: total correct items); Memory (Memory delay-List of Word: total recalled words; Number-Vowel Sequencing: total correct items); Reasoning (Series of Semantic: total correct items; Series of Logic: total correct items); Planning (total number of movements to successfully complete task). *significant level at p < .05 ** significant level at p < .001.

## Discussion

This paper presents a software application for the elderly to tackle the challenge of cognitive decline. Other authors’ results [6] showed that the onset of ADL deterioration can be delayed with cognitive computer training. For this purpose, we developed our PESCO software application and conducted two independent studies to check three factors that play a key role in this context: usability, validation and effectiveness.

Although usability guidelines were considered during PESCO development, direct observation and user comments were obtained from the first method of Study 1 in order to verify PESCO’s usability. The results obtained with this method, however, revealed that certain usability aspects should be more restrictive. New solutions should also be added to the interface to solve any interaction or comprehension difficulty that was not previously considered in the current version of PESCO.

The following modifications should be made: (1) the use of a larger font in the figures; (2) the inclusion of both an audio version to make the application more accessible and a control for adjusting the volume and sound frequency; (3) the addition of borders and colors to make selectable buttons and selections performed more distinguishable; (4) the simplification and abbreviation of certain instructions and the elimination of distracters; (5) completion of several demos and trial runs to ensure that users fully understand the instructions; (6) fine tuning of the difficulty levels, mainly in terms of speed, expected timing and number of items supplied and presented.

We also noticed that the mode of interaction of one of the exercises should be changed. Since some of the participants required alternative interaction devices such as keyboards with large keys, buttons and touch screen buttons, each person’s needs should be studied beforehand in order to provide the most suitable resources.

In terms of the method for evaluating usability (questionnaires), the result of analysis of the user responses indicates that participants were able to overcome their difficulties (Item 1) and initial frustration (Item 2) with PESCO as the sessions progressed. Through gradual contact with the tool, participants learned how to use buttons and there was an improvement in how instructions and other messages were read and understood. Almost 80% of the users did not feel frustrated after the first session and found the PESCO exercises to be both enjoyable (Item 6) and useful (Item 7). This percentage had risen to 97.8% by the end of the twelve sessions. This was the first time that most of the participants had used a computer. Believing that PESCO might improve their cognitive skills, they were able to overcome their fear of technology.

Results from concurrent validation showed that all assessment tests shared the same main construct (attention, memory, reasoning and planning) as the standardized tests used to find a correlation relationship. Several authors warn that there is often a low correspondence between pen-and-paper and computerized assessment [27,28]. In this study, the correlation between construct components was moderate and statistically significant. These results are important since they indicate that PESCO allows us to estimate a person’s cognitive status in computerized form. Validated assessment tests in PESCO also enable the changes following programmed training sessions to be measured.

PESCO’s cognitive stimulation activities have proved effective for improving attention, working memory and planning skills. Better performance in the people who followed the PESCO cognitive training module is not due to using computers since there was no such improvement in the skills of the control group. It is, however, necessary to ascertain whether such improvements will be maintained in the long term when users perform their ADL. In order to continue current research, we are developing a new tool which uses virtual and augmentative reality to assess and train IADL. This new software is called VIRTRA-EL (Virtual Training for the Elderly) and uses all the PESCO tests and exercises. The main advantages of this inclusion are that it supports real-time supervision by the therapist, improving and encouraging group activities and communication between the elderly and their carers, and incorporating other kinds of activities related to physical activity and nutrition. This will allow a large database to be obtained by automatically collecting data from the exercises performed by each user on a server. Using this data, longitudinal studies into cognitive impairment factors and predictions may then be conducted. Since we assessed the usability of PESCO tests and exercises in our current study, their accessibility could be evaluated in future work using the new software.

While there are many software applications in English and Spanish (e.g. *CogRehab*[29], *Rehacom*, *Cogniplus and Vienna Test System*[30], *Reeduca*[31], *Entrenador Personal*[32], *Smartbrain*[33] or *Gradior*[34]), they are expensive, or at least not free, and most provide no evidence of construct validity or effectiveness. The benefits of an open-source application such as PESCO are its reliability, stability, auditability, low cost, flexibility and freedom for adaptation to other languages and cultures, and its support facility. An open-source application can be used and maintained by a large group of therapists and in this case developers.

We are currently working on the inclusion of the exercises validated in PESCO on the Internet VIRTRA-EL Platform as a module of its architecture.

The main limitations of this study are its small sample size, lack of any follow-up of improvements in participants and lack of traditional standard evaluation in the post-stimulation time of assessment. Furthermore, the short duration of PESCO’s cognitive stimulation might not be sufficient for improvements in verbal memory and reasoning functions. Since we do not know whether the control group also showed changes in usability as a result of using the computer during the intervention phase, it is possible that changes in the performance in cognitive tasks might be related to changes in usability.

## Conclusions

PESCO has been designed following usability guidelines for the elderly. During the study, however, participants found that certain aspects of the interface of some tests were easy to use and understand. We are working on a new version of PESCO within the VIRTRA-EL project (http://asistic.ugr.es/virtra-el), which provides a web platform and includes the usability improvements from this study. The PESCO cognitive test module has demonstrated its concurrent validity with traditional cognitive evaluation tests. Meanwhile, its stimulation module is effective for improving attention, working memory and planning skills. We provide an open-source tool which may be freely distributed and adapted and used both at home and in elderly care homes, educational, health and social centers.

## Competing interests

The authors declare that they have no competing interests.

## Authors’ contribution

SS-R and AC designed the study and the PESCO tool. SS-R and SR-P collected data and performed the statistical analysis. MVH and MJR-F were responsible for developing the PESCO tool and collaborated with the design. All the authors read and approved the final manuscript.
